# Serum neurofilament light chain predicts cognitive worsening in secondary progressive multiple sclerosis better than brain MRI measures

**DOI:** 10.1177/13524585221122916

**Published:** 2022-09-20

**Authors:** Lorenzo Gaetani, Menno M Schoonheim

**Affiliations:** Section of Neurology, Department of Medicine and Surgery, University of Perugia, Perugia, Italy; Department of Anatomy and Neurosciences, MS Center Amsterdam, Amsterdam Neuroscience, Amsterdam UMC, Vrije Universiteit Amsterdam, Amsterdam, The Netherlands

Cognitive impairment (CI) is now fully accepted as a clinical manifestation of multiple sclerosis (MS). It affects people with MS since the earliest phases of the disease, and it can worsen over time like other functional systems, especially in progressive MS.^1^ Mechanisms underlying CI are complex, mainly thought to be based on the accumulation of structural disconnection, neurodegeneration and failure of functional reserve capacity.^2^ Given this hypothesized complex, slow cascade leading to CI, preserving cognitive functioning might still be in the therapeutic window once a diagnosis of secondary progressive MS (SPMS) is made based on the evidence of progression in other neuronal domains.^3^ As more data are accumulating on disease modifying therapies showing an effect on cognition and more trials are being designed, it is now necessary to identify and validate markers useful to stratify patients at risk, monitor and predict the course of CI in MS.

Neurofilament light chain (NfL) has proven to be a reliable marker of acute axonal injury,^4^ driven by active inflammatory lesions in MS, which supports its use to monitor inflammatory demyelinating lesion activity with axonal damage.^5^ In MS, cerebrospinal fluid and serum NfL (sNfL) have already been demonstrated to cross-sectionally associate with the severity of CI, and sNfL has shown to predict its worsening in SPMS.^6,7^ However, is not yet clear whether NfL per se is informative with regard to cognition in MS, or just duplicates information that can be derived from other techniques, such as information on neurodegeneration from brain magnetic resonance imaging (MRI). In other words, what is the real benefit of sNfL over brain MRI measures in terms of predicting CI in MS, and specifically in SPMS?

This issue of *Multiple Sclerosis Journal* (MSJ) features a study investigating the predictive role of sNfL, brain MRI T2 lesion volume and normalized regional volumes for cognitive worsening in people with SPMS.^8^ Williams et al. utilized data from the phase 2 randomized, double-blind placebo-controlled MS-STAT trial that evaluated the effects of simvastatin in SPMS.^9^ During this trial, people with SPMS underwent a thorough neuropsychological assessment at baseline, month 12 and month 24. The battery focused mainly on the Wechsler Abbreviated Scale of Intelligence (WASI), but also included additional tests relevant for neurodegenerative disease. At baseline, month 12 and 25, people with MS underwent brain MRI according to a standardized protocol, and at baseline, month 6, 12 and 24, they also underwent blood samplings. The study presented in this MSJ issue aimed to primarily assess the relationship between baseline sNfL and the change in WASI Full Scale Intelligence Quotient (IQ) over 2 years, while adjusting for baseline demographic and MRI variables. Data were analysed for 110 people with SPMS participating to the trial with sufficient sNfL, brain MRI and neuropsychometric data to be included in the primary analysis.

The main finding of this study was that sNfL was predictive of subsequent cognitive decline, while MRI measures were not. A doubling of baseline sNfL was associated with a 0.010 [0.003–0.017] point per month faster decline in WASI Full Scale IQ Z-score (*p* = 0.008), when adjusting for all relevant MRI measures. Therefore, each doubling of baseline sNfL corresponded to a 0.24 [0.07–0.41] point decline in WASI Full Scale IQ Z-score over the 2 years of follow-up of the study. Cross-sectionally, at baseline, CI was not related to sNfL levels, but did show effects for higher brain T2 lesion volume and lower normalized cortical deep grey matter and transverse temporal gyrus volumes.

The results of the study are important as the different associations shown by sNfL and brain MRI measures with cognitive function at baseline and during follow-up seem to indicate a certain order of events that can be monitored and perhaps treated. The authors hypothesize that lesion volumes and quantifications of neurodegeneration represent the sum of all pathophysiological processes occurred in the past. As a result, the lower the normalized volumes and the greater the T2 lesions volume at baseline, the worse the cognitive performance on a cross-sectional association. On the contrary, sNfL is a dynamic measure of the ongoing axonal injury, with its levels reflecting the magnitude of pathophysiological mechanisms linked to MS in the previous 3 months. The extent of recent axonal damage measured by baseline sNfL could therefore represent how severely patients are at risk of developing new lesions and neurodegeneration, and hence subsequent CI in the following 2 years.

The study by Williams et al. has the merit of having focused on the population of people with SPMS, based on a standardized prospective data collection from a randomized clinical trial, which is a major strength. There are some unresolved questions, however. For instance, gadolinium enhancing lesions have also been shown to predict cognitive decline in SPMS.^7^ Given their overlap in acute inflammatory measurements and the debate on the rationalization of contrast agents in MS, it would be important to study whether sNfL could perhaps even entirely replace contrast agents in SPMS. In addition, it remains to be confirmed whether the predictive potential of sNfL in this study is also seen using (shorter) neuropsychological batteries specifically validated for MS and thus more commonly used in MS clinical practice and research. Earlier work using such batteries has shown a predictive value of MRI in progressive MS,^10^ which could be explained by a more severely affected cohort, as CI was relatively mild in this paper. Finally, if the hypothesis of a specific order of events leading to CI is to be proven, that is, starting with acute neuroaxonal damage, then neurodegeneration, finally leading to a ‘network collapse’,^2^ larger longitudinal cohorts are now needed, including data from the relapsing remitting stage as well as the transition from relapsing remitting MS towards SPMS.

In summary, this study shows that sNfL provides a reliable prognostic evaluation on subsequent cognitive decline in people with SPMS. We would now encourage the field to further validate and improve its use in this specific population, both in the context of clinical trials and of clinical practice.
